# Identification of two novel bullous pemphigoid- associated alleles, HLA-DQA1*05:05 and -DRB1*07:01, in Germans

**DOI:** 10.1186/s13023-021-01863-9

**Published:** 2021-05-19

**Authors:** Christian Schwarm, Damian Gola, Maike M. Holtsche, Anabelle Dieterich, Anita Bhandari, Miriam Freitag, Peter Nürnberg, Mohammad Toliat, Wolfgang Lieb, Michael Wittig, André Franke, Margitta Worm, Michael Sticherling, Jan Ehrchen, Claudia Günther, Regine Gläser, Wiebke K. Peitsch, Miklós Sárdy, Rüdiger Eming, Michael Hertl, Sandrine Benoit, Matthias Goebeler, Claudia Pföhler, Manfred Kunz, Alexander Kreuter, Nina van Beek, Jeanette Erdmann, Hauke Busch, Detlef Zillikens, Christian D. Sadik, Misa Hirose, Inke R. König, Enno Schmidt, Saleh M. Ibrahim, German AIBD Study Group

**Affiliations:** 1 Lübeck Institute of Experimental Dermatology, University of Lübeck, Lübeck, Germany; 2 CRIS, Center for Research On Inflammation of the Skin, University of Lübeck, Lübeck, Germany; 3 Institute of Medical Biometry and Statistics, University of Lübeck, University Clinic Schleswig-Holstein, Campus Lübeck, Lübeck, Germany; 4 Department of Dermatology, University Hospital Schleswig-Holstein, Campus Lübeck, Lübeck, Germany; 5 Institute for Cardiogenetics, University of Lübeck, Lübeck, Germany; 6 Cologne Center for Genomics, University of Cologne, Cologne, Germany; 7 Institute of Epidemiology and Popgen Biobank, Christian Albrechts-University of Kiel, Kiel, Germany; 8 Institute of Clinical Molecular Biology, Christian Albrechts-University of Kiel, Kiel, Germany; 9 Allegologie Und Immunologie, Klinik Für Dermatologie, Venerologie und Allergologie, Charité Universitätsmedizin Berlin, Berlin, Germany; 10 Department of Dermatology, University Hospital Erlangen, Deutsches Zentrum Immuntherapie (DZI), Erlangen, Germany; 11 Department of Dermatology, University of Münster, Münster, Germany; 12 Department of Dermatology, University Hospital Dresden, Dresden, Germany; 13 University Clinic Schleswig-Holstein, Campus Kiel, Kiel, Germany; 14 Department of Dermatology and Phlebology, Vivantes Klinikum Im Friedrichshain, Berlin, Germany; 15 Department of Dermatology, Venereology and Dermatooncology, Semmelweis University, Budapest, Hungary; 16 Department of Dermatology and Allergology, University Hospital, LMU Munich, Munich, Germany; 17 Department of Dermatology and Allergology, Philipps-Universität Marburg, Marburg, Germany; 18 Department of Dermatology, Venereology and Allergology, University Hospital Würzburg, Würzburg, Germany; 19 Department of Dermatology, Saarland University Medical Center, Homburg/Saar, Germany; 20 Department of Dermatology, Venereology and Allergology, Leipzig University Medical Center, Leipzig, Germany; 21 Department of Dermatology, Venerology and Allergology, Helios St. Elisabeth Hospital Oberhausen, University Witten/Herdecke, Oberhausen, Germany; 22 College of Medicine and Sharjah Institute for Medical Research, University of Sharjah, Sharjah, UAE

**Keywords:** Bullous pemphigoid, Autoimmune blistering skin diseases, GWAS, HLA, Germans

## Abstract

Bullous pemphigoid (BP) is the most common autoimmune skin blistering disease characterized by autoimmunity against the hemidesmosomal proteins BP180, type XVII collagen, and BP230. To elucidate the genetic basis of susceptibility to BP, we performed the first genome-wide association study (GWAS) in Germans. This GWAS was combined with HLA locus targeted sequencing in an additional independent BP cohort. The strongest association with BP in Germans tested in this study was observed in the two HLA loci, HLA-DQA1*05:05 and HLA-DRB1*07:01. Further studies with increased sample sizes and complex studies integrating multiple pathogenic drivers will be conducted.

**Supplementary Information:**

The online version contains supplementary material available at 10.1186/s13023-021-01863-9.

Bullous pemphigoid (BP) is the most common autoimmune skin blistering disease in Europe. BP is characterized by autoimmunity against the hemidesmosomal proteins BP180, type XVII collagen, and BP230 [1]. The pathophysiology of BP is incompletely understood and the genetic basis of susceptibility to BP is largely unknown as large-scale genetic studies have so far been hampered by the low prevalence of the disease.

Therefore, we set out to perform the first genome-wide association study (GWAS) in Germans to identify the gene variants predisposing to BP. For this purpose, 446 BP patients were recruited by the *German AIBD Genetics Study Group* and 433 German age- and sex-matched controls were retrieved from the *Popgen* biobank (Kiel, Germany). The cohorts were genotyped in two batches, both containing patient and control samples, using Applied Biosystems™ UK Biobank Axiom™ Array chips, containing 825,927 markers (Additional file 1: Materials and methods).

The meta-analysis of the GWAS revealed a strong association with SNPs within the HLA locus (6p21.1–21.3) (Additional file 1: Materials and methods; Table 2; Additional file 2: dataset 1), reaching genome-wide significance for 6 SNPs (*p* < 5E−08). In addition to the HLA locus, multiple loci of suggestive association superseding the background noise were identified. A λ-GC of 0.8501081 for the meta-analysis, adjusted for 100 cases and controls, indicates that the results for the non-HLA loci may be conservatively biased and include more false negatives than expected. We therefore focused on the HLA locus for further analysis. Allele calling based on the raw GWAS data (Batch 1 and Batch 2, as a discovery study) showed 18 HLA alleles that are associated with BP (*p* < 0.05), including DQA1*05:05 (*p* = 1.23189E−08), DQB1*03:01 (*p* = 1.10574E−05) and DRB1*07:01 (*p* = 0.000236558; Additional file 3: dataset 2). To confirm these findings, the entire HLA locus was deep sequenced in 87 independent BP patients samples and analysed with reference to a northern German blood donor cohort (n = 547), coded by the National Marrow Donor Program (NMDP) standard as a replication cohort (Additional file 1: Materials and methods; Additional file 3: dataset 2). A meta-analysis of the discovery and the replication cohorts revealed that two of 18 HLA alleles identified in the discovery study, HLA-DQA1*05:05 and -DRB1*07:01, were confirmed (Table 1).

Of the identified HLA alleles, the association of DQA1*05:05 (*p* = 8.9783E−7; Table 1) is in line with previous reports: it was identified in Brazilian [2] and Chinese [3] BP cohorts as a BP-susceptible allele. The HLA-DRB1 gene allele DRB1*07:01 was previously identified as a protective allele in Chinese population [3]. However, these reports were based on studies using small number of non-European cohorts. These alleles have not been reported in Germans or other European BP patients, to the best of our knowledge. The allele HLA-DRB1*07:01 has been reported to be associated with increased susceptibility to systemic lupus erythematosus and with the production of autoantibodies (anti-Sm) in Koreans [4].

Interestingly, the allele DQA1*05:05 is reported to be in linkage disequilibrium with the allele DQB1*03:01, which is reportedly associated with BP in multiple ethnic backgrounds including Caucasians [2, 5–7], in different populations [8, 9]. The functional impact of the DQB1*03:01 has been well documented [10, 11] as well as its strong association with drug-induced BP [12]. When the conditional analysis was performed in our data, DQA1*05:05 is conditional on DQB1*03:01 and vice versa (Additional file 4: dataset 3). Even though these alleles are significantly associated with BP in the discovery cohort, the effects of both alleles are not statistically significant at 0.05 under condition of each other allele. This finding supports the linkage disequibrilium of these two alleles. Yet, the confidence interval of the effect of the DQB1*03:01 in the meta-analysis is still strictly positive (Table 1). A similar phenomeno is also observed with the DRB1*11:01 allele, which has also been previously reported to be associated with BP [12, 13], i.e., in significant linkage disequilibrium with HLA-DQA1*05:05 [9], and its effect in the meta-analysis is positive despite its statistical non-significance (Table 1).

As the sample size of our study is comparably smaller for a GWAS compared with today’s standard of GWAS for common diseases, such as cardiovascular diseases, detection of associated variants can only be limited to common variants shared between the patients, i.e., HLA locus. Indeed, the allele frequency of DRB1*07:01 is approximately 1.21265E−1 in Germans [14]. However, considering the rare nature of BP, disease susceptibility may be attributable to rare variants spread across many different genes other than the HLA locus, affecting shared pathways. These gene variants would therefore only possess a small effect size and weak associations, which has in recent times been characterized as a defining feature of GWA studies, accounting for what is occasionally referred to as ‘missing heritability’ [15]. To address this issue of minor effect variants, which is typical for multifactorial and polygenic disorders, targeted sequencing approaches are currently being employed. Another potential explanation for the lack of significant association outside the HLA locus in this GWAS is the potential involvement of environmental factors (e.g., diet, commensal bacteria) in the pathogenesis of BP. Therefore, the complex gene-environment interactions will be further investigated by the *German AIBD Genetics Study Group*.

In conclusion, we performed the very first GWAS in BP using the largest cohort in the world. Together with the HLA locus targeted sequencing result in an additional independent BP cohort, the strongest association with BP in Germans tested in this study was observed in the HLA loci, HLA-DQA1*05:05 and HLA-DRB1*07:01. However, further studies using increased sample sizes and complex studies integrating multiple pathogenic drivers will be conducted.

**Table 1:** HLA alleles associated with BP identified in this study

Gene	Allele	Study	P-value	95% CI [lower, upper]	P-value (Hommel adjusted)	OR	OR [lower, upper]
A	A*03:01	Discovery	0.034710658	[0.0208, 0.5591]	0.99908782	1.342686047	[1.0279, 1.7538]
		Replication	0.58697531	[− 2.3684, 2.4882]			
		Meta-analysis	0.030601855	[0.0276, 0.5618]			
B	B*08:01	Discovery	0.02405483	[− 1.0689, − 0.0751]	0.354370169	0.571262096	[0.3987, 0.8184]
		Replication	0.122675915	[− 1.6824, 0.1457]			
		Meta-analysis	0.002271604	[− 0.9194, − 0.2004]			
	B*37:01	Discovery	0.014767834	[− 2.1156, − 0.2300]	0.99908782	0.309496161	[0.1206, 0.7946]
		Replication		[NA, NA]			
		Meta-analysis	0.014767834	[− 2.1156, − 0.2300]			
	B*38:01	Discovery	0.034632674	[0.0562, 1.4982]	0.99908782	2.265753431	[1.1271, 4.5546]
		Replication	0.31618692	[− 1.8167, 4.6785]			
		Meta-analysis	0.021680056	[0.1197, 1.5161]			
C	C*04:01	Discovery	0.001280217	[0.2229, 0.9162]	0.421598674	1.637652525	[1.1862, 2.2609]
		Replication	0.996052508	[− 0.9515, 0.8322]			
		Meta-analysis	0.002719991	[0.1708, 0.8158]			
	C*06:02	Discovery	0.008418861	[− 0.7650, − 0.1124]	0.99908782	0.681311521	[0.5036, 0.9218]
		Replication	0.900089924	[− 0.9435, 0.6911]			
		Meta-analysis	0.012845693	[− 0.6860, − 0.0814]			
DQA1	DQA1*02:01	Discovery	0.000236558	[− 1.0487, − 0.3194]	0.99908782	0.585901665	[0.3874, 0.8860]
		Replication	0.821096838	[− 1.0133, 0.6346]			
		Meta-analysis	0.011292202	[− 0.9482, − 0.1210]			
	DQA1*05:01	Discovery	0.01391165	[− 1.8228, − 0.2060]	0.99908782	0.454746049	[0.2362, 0.8757]
		Replication	0.710972174	[− 1.1973, 0.6923]			
		Meta-analysis	0.01842072	[− 1.4433, − 0.1328]			
	DQA1*05:05	Discovery	1.23189E-08	[0.5081, 1.0413]	**8.97827E**−**07**	**2.04569062**	**[1.6082, 2.6022]**
		Replication	0.109203061	[− 0.1211, 1.0059]			
		Meta-analysis	5.54214E-09	[0.4751, 0.9564]			
DQB1	DQB1*02:01	Discovery	0.01391165	[− 1.8228, − 0.2060]	0.109465545	0.401052978	[0.2366, 0.6797]
		Replication	0.167679623	[− 1.9823, 0.2065]			
		Meta-analysis	0.000688463	[− 1.4413, − 0.3861]			
	DQB1*02:02	Discovery	0.00278635	[− 1.0331, − 0.2150]	0.630186648	0.581980108	[0.4020, 0.8426]
		Replication	0.70501071	[− 1.1817, 0.6072]			
		Meta-analysis	0.004145965	[− 0.9114, − 0.1712]			
	DQB1*03:01	Discovery	1.10574E−05	[0.4521, 1.1798]	0.99908782	1.835883195	[1.0057, 3.3513]
		Replication	0.889954019	[− 0.4514, 0.5064]			
		Meta-analysis	0.047871422	[0.0057, 1.2094]			
DRB1	DRB1*03:01	Discovery	0.00383576	[− 1.5352, − 0.2947]	0.99908782	0.233278746	[0.2608, 0.9996]
		Replication	0.96806271	[− 0.8528, 0.8063]			
		Meta-analysis	0.049872143	[− 1.3439, − 0.0004]			
	DQB1*06:01	Discovery	0.032188411	[− 2.7873, − 0.1237]	0.99908782	0.510607993	[0.0616, 0.8836]
		Replication		[NA, NA]			
		Meta-analysis	0.032188411	[− 2.7873, − 0.1237]			
	DRB1*07:01	Discovery	0.000236558	[− 1.0487, − 0.3194]	**0.01463864**	**0.51542337**	**[0.3698, 0.7183]**
		Replication	0.170712241	[− 1.4540, 0.1740]			
		Meta-analysis	9.09232E-05	[− 0.9947, − 0.3308]			
	DRB1*11:01	Discovery	0.011897502	[0.1177, 0.9489]	0.069015473	1.627822652	[1.2409, 2.1354]
		Replication	0.176829458	[− 0.2289, 1.1130]			
		Meta-analysis	0.00043406	[0.2158, 0.7587]			
	DRB1*12:01	Discovery	0.00440014	[0.4163, 2.2540]	0.99908782	3.18164846	[1.3614, 7.4358]
		Replication	0.914702144	[− 2.8669, 2.0659]			
		Meta-analysis	0.007535421	[0.3085, 2.0063]			
	DRB1*15:02	Discovery	0.032188411	[− 2.7873, − 0.1237]	0.99908782	0.233278746	[0.0616, 0.8836]
		Replication		[NA, NA]			
		Meta-analysis	0.032188411	[− 2.7873, − 0.1237]			

Numbers in bald, statistically significant value in the meta-analysis

*OR* odds ratio, *CI* confidence interval

## Supplementary Information

**Additional file 1**. Materials and methods. References. Table 1: The age range of samples used in this study. Table 2: Sample numbers of the BP GWAS cohorts. Table 3: Distribution of posterior probabilities of imputed HLA alleles.

**Additional file 2**. Dataset 1. Meta-analysis results data table of suggestive SNPs (-log10(p) > 5.0).

**Additional file 3**. Dataset 2. Full HLA sequencing data table.

**Additional file 4**. Dataset 3. HLA sequencing “conditional” analysis data list.

**Additional file 5**. Dataset 4. Imputation accuracy table.

## Data Availability

Datasets related to this article were submitted to the European Genome-phenome Archive (EGA) with ID: EGAD00010001956 (GWAS Called Data Batch 2 Called genotypes of samples in batch 2 of CRU303 GWAS), EGAD00010001955 (GWAS Raw Data Batch 1 Controls Raw data files of samples in batch 1 of CRU303), EGAD00010001954 (GWAS Raw Data Batch 1 Cases Raw data files of samples in batch 1 of CRU303 GWAS), EGAD00010001953 (GWAS Raw Data Batch 2 Controls Raw data files of samples in batch 2 of CRU303), EGAD00010001952 (GWAS Raw Data Batch 2 Cases Raw data files of samples in batch 2 of CRU303 GWAS), and EGAD00010001951 (GWAS Called Data Batch 1 Called genotypes of samples in batch 1 of CRU303 GWAS). These data are available upon request.
