# Supplementary material for: Identification of two novel bullous pemphigoid- associated alleles, HLA-DQA1*05:05 and -DRB1*07:01, in Germans
Source: Orphanet J Rare Dis. 2021 May 19;16:228. doi: 10.1186/s13023-021-01863-9 (PMC8136166; doi:10.1186/s13023-021-01863-9)
Supplement: Supplementary file 1 — Additional file 1. Materials and methods. References. Table 1: The age range of samples used in this study. Table 2: Sample numbers of the BP GWAS cohorts. Table 3: Distribution of posterior probabilities of imputed HLA alleles. [file 13023_2021_1863_MOESM1_ESM.docx]

**Orphanet Journal of Rare Diseases**

**Full title:**

**Identification of three novel bullous pemphigoid- associated alleles, HLA-DQA1*05:05 and -DRB1*07:01 in Germans.**

**Additional file 1:**

Supplementary materials and methods

Supplementary references

Supplementary table 1: The age range of samples used in this study

Supplementary table 2: Sample numbers of the BP GWAS cohorts

Supplementary table 3: Distribution of posterior probabilities of imputed HLA alleles

**Additional file 1**

**Supplementary materials and methods**

*Study cohorts*

446 BP patients were recruited by the *German Autoimmune Bullous Disease Study Group.* Diagnosis of BP was based on (i) a compatible clinical phenotype without predominant mucosal lesions, (ii) linear deposits of IgG and/or C3 at the basal membrane zone by direct immunofluorescence microscopy of a perilesional biopsy, and (iii) serum IgG antibodies that labelled the epidermal side of the artificial split by indirect immunofluorescence microscopy of human salt-split skin or reactivity by BP180 NC16A by ELISA (Euroimmun, Lübeck, Germany; MBL, Nagoya, Japan) [1,2]. EDTA blood was stored at ‑80°C until processed. DNA was extracted using the QIAamp DNA Maxi Blood Kit (Qiagen) and stored at ‑80°C until genotyping. 433 German age- and sex-matched controls were retrieved from the biobank *Popgen* (Kiel, Germany) and used as controls.

The age range of samples used in this study is summarized in the **Supplemental table 1**. In short, the age in BP patients was 76.8 years on average plus/minus 10.7 (males) and 75.9 years on average plus/minus 12.3 (females), and those in controls were 71.8 years on average plus/minus 5.7 (males) and 72.7 years on average plus/minus 4.7 (females). The table below summarizes the age range of samples used in this study.

The study was performed following the Declaration of Helsinki and approved by the ethics committee of the University of Lübeck (12-178 and 15-051) and the corresponding local ethics committees of the study centers.

*Genome-wide association study, quality control, PC analysis, and identification of outliers*

Samples were genotyped in two batches due to the sample recruitment timing, both containing patient and control samples (**Supplementary table 2**), on Applied Biosystems™ UK Biobank Axiom™ Array chips, containing 825,927 markers (Thermo Fisher Scientific, Bremen, Germany)[3]. The calling of the genotypes was performed according to the manufacturer’s best practice guidelines [4]. Quality control and association analyses were performed in each batch separately. To this end, SNPs with a minor allele frequency (MAF) below 0.05, 2% missing rate or with a *P* value in a test for deviation from the Hardy-Weinberg equilibrium with mid-p adjustment in controls less than 0.0001 were removed from the analysis. Samples were excluded if the genotype call rate was less than 95%, the heterozygosity rate exceeded three standard deviations from the mean heterozygosity rate or the kinship coefficient $\hat{\pi}$was greater than 0.1875. All quality control steps were performed using plink 1.9b3.45 [5]. Finally, individuals identified as outliers by smartpca from EIGENSOFT 6.1.4 [6–8] were also excluded. Missing sex was imputed using the ratio of the average probe intensity of nonpolymorphic probes on the Y chromosome to the average probe intensity of nonpolymorphic probes on the X chromosome. If the ratio was less than 0.54, the sex was imputed as female, and if it was greater than 1.0, the sex was imputed as male. After exclusion of samples, the criteria on SNP level were applied again, resulting in a final set of 386296 SNPs, 287 cases, and 383 controls for batch 1, and 384773 SNPs, 151 cases, and 34 controls for batch 2.

*Imputation*

To prepare the batches for imputation, we used GenotypeHarmonizer 1.4.18 [9] to ensure correct strand orientation and to synchronize variant identifier names with the imputation reference panel. The 2013/05/02 release of the 1000 Genomes project [10] was used as reference for genotype harmonization.

Phasing of the chromosomes was done using shapeit v2.r837.GLIBCv2.12 [11] with 10 burn-in, 10 pruning and 50 main iterations. The number of conditioning states for haplotype estimation was set to 200 with a window size of 2 mega bases. To be compliant with IMPUTE2, we used a universal effective population size of 20000. IMPUTE2 2.3.2 [12,13] was applied to intervals of 2.5 mega bases with buffer regions set to 1 mega bases. The 1000 Genomes haplotypes – Phase 3 integrated variant set release in NCBI build 37 (hg19) coordinates was used as reference panel.

*Genome-wide association analysis*

Genome-wide association testing was done using snptest 2.5.2 [14] adjusting for sex using a logistic regression model with an additive coding for the SNPs. A meta-analysis of the two batches was performed using meta 1.7 [14] under the assumption of a random effects model, and only included SNPs with a MAF > 0.05 in both batches and an imputation info score > 0.6. As an additional QC step, the variant output was subsequently checked against the Varsome database, and variants failing the MAF threshold of 0.05 in the gnomeAD annotation were removed from the list.

*HLA sequencing, HLA-allele calling, and data analysis*

HLA alleles were estimated based on quality controlled pre-imputed SNP genotypes from the GWAS using the R package HIBAG [15]. HIBAG provides pre-trained random forest models to predict the HLA genotype posterior probabilities based on SNP genotypes (**Supplementary table 2**). We used the publicly available model trained on samples of European ancestry (<http://public-html.biostat.washington.edu/~bsweir/HIBAG/param/European-HLA4-hg19.RData>).

Two hundred BP samples used in GWAS and additional independent 88 BP samples were deep sequenced for the HLA locus. The DNA library was prepared using TruSight HLA v2 sequencing panel (Illumina Inc.), and sequenced on an Illumina HiSeq4000 platform with 150 base-pair paired-end reads.

A primary HLA-calling was successfully achieved for 281 of 286 samples, using the two algorithms HD-Seq [16] and STC-Seq [17]. Discordant allele calls were singled out (n = 339, 3.6 %). These discordant calls were reduced to distinction at the protein level (n = 203, 2.2 %), as divergence at the cDNA level was not expected to yield functional differences at the antigen presentation level. These remaining divergent calls were subsequently visually inspected via the tertiary call algorithm HLAssign [18], utilizing the forward and reverse FASTQ files for each sample. Calls were made based on sequence coverage, alignment quality and SNP presence.

Control data were derived from 548 healthy, northern German blood donors, available at the Popgen Biobank ([www.epidemiologie.uni-kiel.de/biobanking/biobank-popgen](http://www.epidemiologie.uni-kiel.de/biobanking/biobank-popgen)). In contrast to the patient samples, control samples were not genotyped for the HLA genes *E, F, G, H, J, K, L, T, V, W, Y*. Final calls were supplied in the form of total calls per genotype, with ambiguous calls annotated following National Marrow Donor Program (NMDP) guidelines [19]. Due to these ambiguities, analysis on second level HLA allele level is the most convenient. NMDP standard codes from 2020/03/02 were used to resolve the ambiguities and HLA allele frequencies were obtained from previous report [20]. To resolve ambiguities in the observed genotypes of controls, genotype frequencies were calculated based on the allele frequencies assuming Hardy-Weinberg equilibrium. HLA alleles with ambiguities were merged with the NMDP code list to get all possible alleles coded by the respective ambiguity code. All HLA alleles were truncated to second level in BP patients and healthy controls. The calculated genotype frequencies were merged based on the resolved and truncated observes HLA alleles. To get the estimated number of controls with resolved HLA alleles, missing allele frequencies were first replaced with the machine eps. Then, the allele frequencies were transformed to reflect the proportion between the observed alleles but sum up to 1. Finally, the observed number of healthy controls was multiplied by the transformed number to get the estimated number of healthy controls for each possible HLA allele with ambiguity. Due to truncation to second level HLA alleles, ambiguities could have been resolved to the same HLA allele, i.e., A*01:CRY resolves to A*01:01:01 and A*01:01:04N and both are truncated to A*01:01. Number of healthy controls were aggregated on resolved and truncated HLA allele level.

For each HLA gene and allele, the number of cases and controls having either 0, 1, or 2 alleles was used in a logistic regression model to model the additive effect of the respective allele in each dataset (GWAS batch 1, GWAS batch 2 and HLA sequencing). To estimate the combined effect of HLA alleles in batches 1 and 2 as discovery set, we utilized a random effects meta-analysis using the R package meta. Considering the data from the HLA sequencing as replication, we estimated the combined effect of HLA alleles in batch 1, 2 and replication by a second meta-analysis.

**Supplementary references**

1. Feliciani C, Joly P, Jonkman MF, Zambruno G, Zillikens D, Ioannides D, et al. Management of bullous pemphigoid: the European Dermatology Forum consensus in collaboration with the European Academy of Dermatology and Venereology. Br J Dermatol. 2015;172:867–77.

2. Schmidt E, Goebeler M, Hertl M, Sárdy M, Sitaru C, Eming R, et al. S2k guideline for the diagnosis of pemphigus vulgaris/foliaceus and bullous pemphigoid. J Dtsch Dermatol Ges. 2015;13:713–27.

3. Affymetrix Inc. UK Biobank Axiom Array, Advancing human health studies with the most powerful genotyping technology, Data Sheet, P/N: GGNO03529, Rev. 1 [Internet]. 2014. Available from: http://www.ukbiobank.ac.uk/wp-content/uploads/2014/04/UK-Biobank-Axiom-Array-Datasheet-2014-1.pdf

4. Affymetrix Inc. Axiom Genotyping Solution, Data Analysis Guide, P/N 702961, Rev.5 [Internet]. 2017. Available from: https://assets.thermofisher.com/TFS-Assets/LSG/manuals/axiom_genotyping_solution_analysis_guide.pdf

5. Purcell S, Neale B, Todd-Brown K, Thomas L, Ferreira MAR, Bender D, et al. PLINK: a tool set for whole-genome association and population-based linkage analyses. Am J Hum Genet. 2007;81:559–75.

6. Galinsky KJ, Loh P-R, Mallick S, Patterson NJ, Price AL. Population Structure of UK Biobank and Ancient Eurasians Reveals Adaptation at Genes Influencing Blood Pressure. Am J Hum Genet. 2016;99:1130–9.

7. Galinsky KJ, Bhatia G, Loh P-R, Georgiev S, Mukherjee S, Patterson NJ, et al. Fast Principal-Component Analysis Reveals Convergent Evolution of ADH1B in Europe and East Asia. Am J Hum Genet. 2016;98:456–72.

8. Price AL, Patterson NJ, Plenge RM, Weinblatt ME, Shadick NA, Reich D. Principal components analysis corrects for stratification in genome-wide association studies. Nat Genet. 2006;38:904–9.

9. Deelen P, Bonder MJ, van der Velde KJ, Westra H-J, Winder E, Hendriksen D, et al. Genotype harmonizer: automatic strand alignment and format conversion for genotype data integration. BMC Res Notes. 2014;7:901.

10. 1000 Genomes Project Consortium, Auton A, Brooks LD, Durbin RM, Garrison EP, Kang HM, et al. A global reference for human genetic variation. Nature. 2015;526:68–74.

11. Delaneau O, Marchini J, 1000 Genomes Project Consortium, 1000 Genomes Project Consortium. Integrating sequence and array data to create an improved 1000 Genomes Project haplotype reference panel. Nat Commun. 2014;5:3934.

12. Howie B, Fuchsberger C, Stephens M, Marchini J, Abecasis GR. Fast and accurate genotype imputation in genome-wide association studies through pre-phasing. Nat Genet. 2012;44:955–9.

13. Howie BN, Donnelly P, Marchini J. A flexible and accurate genotype imputation method for the next generation of genome-wide association studies. PLoS Genet. 2009;5:e1000529.

14. Marchini J, Howie B. Genotype imputation for genome-wide association studies. Nat Rev Genet. 2010;11:499–511.

15. Zheng X, Shen J, Cox C, Wakefield JC, Ehm MG, Nelson MR, et al. HIBAG--HLA genotype imputation with attribute bagging. Pharmacogenomics J. 2014;14:192–200.

16. Kawaguchi S, Higasa K, Yamada R, Matsuda F. Comprehensive HLA Typing from a Current Allele Database Using Next-Generation Sequencing Data. Methods Mol Biol. 2018;1802:225–33.

17. Jiao Y, Li R, Wu C, Ding Y, Liu Y, Jia D, et al. High-sensitivity HLA typing by Saturated Tiling Capture Sequencing (STC-Seq). BMC Genomics. 2018;19:50.

18. Wittig M, Anmarkrud JA, Kässens JC, Koch S, Forster M, Ellinghaus E, et al. Development of a high-resolution NGS-based HLA-typing and analysis pipeline. Nucleic Acids Res. 2015;43:e70.

19. Bray RA, Hurley CK, Kamani NR, Woolfrey A, Müller C, Spellman S, et al. National marrow donor program HLA matching guidelines for unrelated adult donor hematopoietic cell transplants. Biol Blood Marrow Transplant. 2008;14:45–53.

20. Eberhard H-P, Schmidt AH, Mytilineos J, Fleischhauer K, Müller CR. Common and well-documented HLA alleles of German stem cell donors by haplotype frequency estimation. HLA. 2018;92:206–14.

**Supplementary table 1: The age range of samples used in this study**

|  | BP | | | Control | | |
| --- | --- | --- | --- | --- | --- | --- |
|  | Male | Female | Total | Male | Female | Total |
| Minimum age | 15 | 13 | 13 | 42 | 66 | 42 |
| Median age | 78 | 78 | 78 | 73 | 71 | 72 |
| Maximum age | 97 | 96 | 97 | 89 | 90 | 90 |
| Mean age | 76.8 | 75.9 | 76.2 | 71.8 | 72.7 | 72.3 |
| Standard deviation | 10.7 | 12.3 | 11.6 | 5.7 | 4.7 | 5.2 |

**Supplementary table 2: Sample numbers of the BP GWAS cohorts**

| QC | Batch | Status | Male | Female | Unknown | Total |
| --- | --- | --- | --- | --- | --- | --- |
| Pre | 1 | BP | 114 | 178 | 0 | 292 |
|  |  | Control | 155 | 236 | 0 | 391 |
|  | 2 | BP | 64 | 65 | 25 | 154 |
|  |  | Control | 32 | 8 | 2 | 42 |
| Post | 1 | BP | 112 | 175 | 0 | 287 |
|  |  | Control | 151 | 232 | 0 | 383 |
|  | 2 | BP | 72 | 79 | 0 | 151 |
|  |  | Control | 25 | 9 | 0 | 34 |

QC, quality control.

Supplementary table 3: Distribution of posterior probabilities of imputed HLA alleles.

| Batch | HLA Gene | [0,0.25] | [0.25,0.5] | [0.5,0.75] | [0.75,1] |
| --- | --- | --- | --- | --- | --- |
| 1 | A | 1 (0.1%) | 7 (1.0%) | 36 (5.4%) | 626 (93.4%) |
| 1 | B | 5 (0.7%) | 36 (5.4%) | 67 (10.0%) | 562 (83.9%) |
| 1 | C | 2 (0.3%) | 12 (1.8%) | 20 (3.0%) | 636 (94.9%) |
| 1 | DPB1 | 0 (0.0%) | 32 (4.8%) | 123 (18.4%) | 515 (76.9%) |
| 1 | DQA1 | 0 (0.0%) | 13 (1.9%) | 69 (10.3%) | 588 (87.8%) |
| 1 | DQB1 | 0 (0.0%) | 8 (1.2%) | 46 (6.9%) | 616 (91.9%) |
| 1 | DRB1 | 10 (1.5%) | 85 (12.7%) | 203 (30.3%) | 372 (55.5%) |
| 2 | A | 0 (0.0%) | 3 (1.6%) | 11 (5.9%) | 171 (92.4%) |
| 2 | B | 0 (0.0%) | 9 (4.9%) | 13 (7.0%) | 163 (88.1%) |
| 2 | C | 1 (0.5%) | 1 (0.5%) | 4 (2.2%) | 179 (96.8%) |
| 2 | DPB1 | 0 (0.0%) | 3 (1.6%) | 38 (20.5%) | 144 (77.8%) |
| 2 | DQA1 | 0 (0.0%) | 9 (4.9%) | 18 (9.7%) | 158 (85.4%) |
| 2 | DQB1 | 0 (0.0%) | 1 (0.5%) | 15 (8.1%) | 169 (91.4%) |
| 2 | DRB1 | 5 (2.7%) | 24 (13.0%) | 54 (29.2%) | 102 (55.1%) |
